# Pathophysiological Advantages of Spontaneous Ventilation

**DOI:** 10.3389/fsurg.2022.822560

**Published:** 2022-03-14

**Authors:** Judit Lantos, Tibor Németh, Zsanett Barta, Zsolt Szabó, Dóra Paróczai, Endre Varga, Petra Hartmann

**Affiliations:** ^1^Department of Neurology, Bács-Kiskun County Hospital, Kecskemet, Hungary; ^2^Department of Surgery, University of Szeged, Szeged, Hungary; ^3^Institute of Surgical Research, University of Szeged, Szeged, Hungary; ^4^Department of Medical Microbiology, University of Szeged, Szeged, Hungary; ^5^Department of Traumatology, University of Szeged, Szeged, Hungary

**Keywords:** non-intubated, one-lung ventilation, inflammatory response, immune cells, cytokines

## Abstract

Surgical procedures cause stress, which can induce an inflammatory response and reduce immune function. Following video-assisted thoracoscopic surgery (VATS), non-intubated thoracic surgery (NITS) was developed to further reduce surgical stress in thoracic surgical procedures. This article reviews the pathophysiology of the NITS procedure and its potential for reducing the negative effects of mechanical one-lung ventilation (mOLV). In NITS with spontaneous ventilation, the negative side effects of mOLV are prevented or reduced, including volutrauma, biotrauma, systemic inflammatory immune responses, and compensatory anti-inflammatory immune responses. The pro-inflammatory and anti-inflammatory cytokines released from accumulated macrophages and neutrophils result in injury to the alveoli during mOLV. The inflammatory response is lower in NITS than in relaxed-surgery cases, causing a less-negative effect on immune function. The increase in leukocyte number and decrease in lymphocyte number are more moderate in NITS than in relaxed-surgery cases. The ventilation/perfusion match is better in spontaneous one-lung ventilation than in mOLV, resulting in better oxygenation and cardiac output. The direct effect of relaxant drugs on the acetylcholine receptors of macrophages can cause cytokine release, which is lower in NITS. The locoregional anesthesia in NITS is associated with a reduced cytokine release, contributing to a more physiological postoperative immune function.

## Introduction

Surgical procedures, including thoracic surgery, cause stress, which can induce inflammatory responses and reduce the function of the immune system (1). Ventilation applied during surgery can deepen this negative effect (2). These alterations influence the healing of patients after surgery, and more-intensive and longer trauma results in greater surgical stress and an increased inflammatory response (3). To reduce the stress to the patient during thoracic surgery, minimally invasive procedures can be applied. Video-assisted thoracoscopic surgery (VATS) has fewer postoperative complications than thoracotomy (4), and the inflammatory response is reduced after VATS compared to open surgery (5).

Following the innovation and acceptance of VATS, non-intubated thoracic surgery (NITS) was developed to further reduce surgical stress in thoracic surgical procedures (6). It has been shown that the inflammatory response after NITS is lower than that in intubated- and relaxed-surgery cases (7), and it is suggested that NITS might be a least minimally invasive thoracic surgical procedure (8).

**Figure 1:**
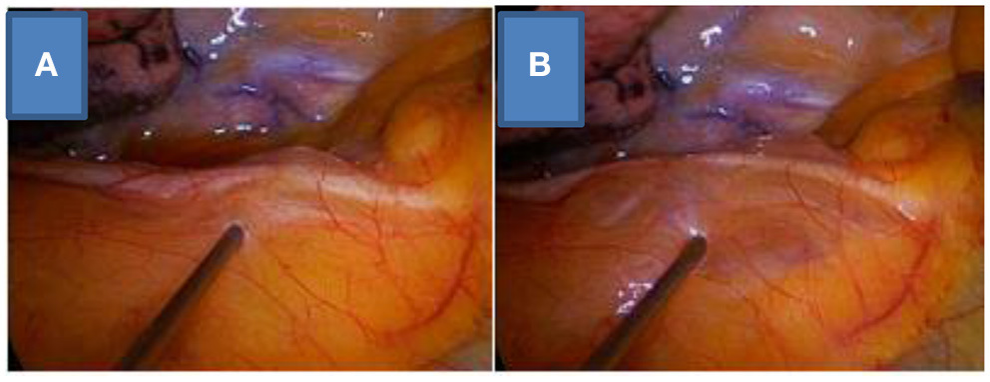
Vagus nerve block on the right side, before **(A)** and after **(B)** the local anesthesia.

**Figure 2:**
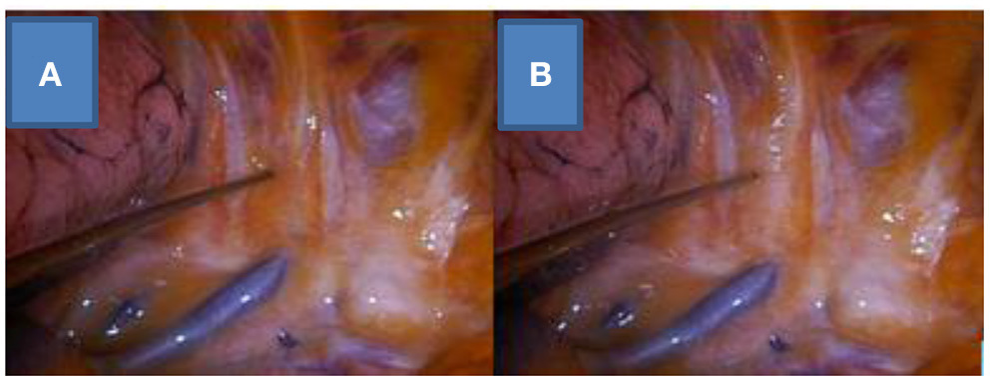
Internal paravertebral/intercostal nerve block on the right side, before **(A)** and after **(B)** the local anesthesia.

NITS can be technically divided into two parts: anesthesia and surgery. During anesthesia, the main difference of the NITS method from the relaxed-surgery method is that in NITS cases, relaxant drugs are not used, the patient is breathing spontaneously during the procedure, and tracheal intubation and mechanical ventilation are not performed. The surgical part of NITS is the same as that in relaxed-surgery cases, but in the internal paravertebral/intercostal + vagus nerve block method, the surgeon administers the local anesthesia (Figures 1, 2).

Two key advantages of NITS that can help reduce surgical stress and inflammatory responses are the non-use of muscle relaxant drugs (9) and the absence of mechanical one-lung ventilation (mOLV) (10). The use of spontaneous ventilation instead of mOLV results in a lower inflammatory response and immune alteration and induces a different pathophysiological state of the cardiorespiratory system.

In this article, the pathophysiological advantages of NITS are discussed in the context of the negative effects of the mOLV procedure, which can potentially be reduced by performing NITS. The immune advantages of NITS are mentioned less thoroughly.

## Effects of mOLV

Mechanical one-lung ventilation and relaxation is currently the recommended procedure for thoracic surgery in cases of lung resection. It is an obligatory process and provides a good view of the patient's anatomy; however, it has negative effects that must be taken into account. The terminology of one-lung ventilation was created in the pre-NITS period, and it is currently classified into two subclasses: mOLV (previously called one-lung ventilation) and spontaneous one-lung ventilation (sOLV). There are many pathophysiological differences between the two methods.

In mOLV, maintaining the oxygen level within or close to the physiological range is achieved by increasing the oxygen level or positive end-expiratory pressure (PEEP) or using ventilation with positive pressure. However, hypoxia develops in 30–90% of patients during mOLV. The parameter settings on the ventilator depend on the experience of the anesthesiologist. The lung alveoli can be damaged if these parameters are non-physiological or intolerable for the lung parenchyma, mainly at the capillary membrane of the alveoli and especially at the endothelial glycocalyx. These changes are the pathophysiological basis of acute lung injury caused by ventilation (11). To reduce or avoid these complications, protective ventilation is recommended, which include keeping the tidal volume to 4–5 ml/kg, ensuring a PEEP of 5–10 cmH_2_O, performing a lung recruitment maneuver, and using volatile anesthesia (12). The application of protective ventilation is easy or manageable in patients with normal lung parenchyma and cardiac function; however, if the patient has obstructive pulmonary disease with pre-existing pulmonary hypertension, protective ventilation can be difficult. Even a slight change in the lung volume in mOLV can cause hypoxia, hemodynamic imbalance, and elevated pulmonary vascular resistance with reduced cardiac output; the correction of the oxygenation requires ventilation with a higher volume and pressure, which can cause injury to the alveoli (13). Clinically, mOLV is similar to the ventilation performed in post-pneumonectomy cases; in a clinical study, an increase of 1 ml/kg of tidal volume in post-pneumonectomy patients was associated with approximately four times more chance of abnormal pathophysiological changes in the lung (14). It can be concluded that the ventilator setting during mOLV, which is used mainly for patients with underlying lung parenchymal disease with low pulmonary capacity, is vulnerable to changes, and a large tidal volume can cause end-inspiratory lung overdistension (volutrauma), which has a high risk of postoperative pulmonary complications (15). Moreover, atelectasis (atelectrauma) may develop in cases of low-volume ventilation, and the abovementioned lung injury (volutrauma) can develop during the correction of the atelectasis. The use of NITS in these patients may be beneficial for preventing these pathophysiological changes.

## Pathological Changes in mOLV

High-volume ventilation and high-pressure ventilation are the main risk factors for damage to the alveoli during mOLV. Elastic and collagen fibers determine the elasticity of the lungs, and the alveolar wall with the alveolar-capillary membrane and endothelial glycocalyx can be ruptured in cases with greater than normal stretch. Lung parenchyma injury can result in cytokine release, recruitment of inflammatory cells (neutrophils, macrophages, and lymphocytes), and edema in the dependent lung (10). In an experimental study, these changes were observed within 90 min from the initiation of mOLV (16). As these immune cells become activated, an inflammatory cascade is induced as a part of the biotrauma of mOLV. In addition, hyperperfusion of the dependent lung develops during mOLV. When hyperperfusion is combined with hyperinflation, alveolar damage occurs, causing interstitial edema and microhemorrhages. Intra-alveolar and inflammatory cells are part of the biotrauma caused by mOLV; other components include cytokine release and inflammatory response reactions (10, 11). As with the other disadvantages of mOLV (volutrauma and atelectrauma), biotrauma can be reduced by performing NITS.

## Physiological Changes in mOLV

Ventilation and perfusion are the most important functions of the lung; due to their close correlation, they are measured together as the ventilation/perfusion (V/Q) ratio. Similar to the pathological changes noted above, the physiological changes during mOLV are caused by damage to the alveoli (volutrauma/barotrauma). A V/Q mismatch can occur during the different periods of the mOLV or sOLV procedure, and many factors influence it (patient position, exploration of the thoracic cavity, and surgical manipulation of the operated lung). The special characteristics of ventilation during mOLV are detailed above. The pulmonary artery, pulmonary vein, and alveolar pressures have a central role in perfusion. In cases of increased lung volume, the alveolar capillaries are compressed, and the pulmonary vascular resistance increases. Hypoxic pulmonary vasoconstriction is the oxygen-sensing mechanism of the lung that reduces perfusion at the hypoxic part of the lung and drives it to a better-ventilated area. The result of these factors is a V/Q mismatch. In an experimental study, mOLV was shown to cause a V/Q mismatch, with hyperperfusion and alveolar damage in the dependent lung (16). The beneficial effect of spontaneous ventilation on the V/Q ratio can be demonstrated in patients with acute respiratory distress syndrome. Spontaneous ventilation increases the ventilation and perfusion rates and improves the heart function and oxygenation (17). The same effect can also be observed in sOLV cases.

The complications of mOLV in the dependent lung can disappear or diminish in NITS with sOLV, but other emerging disadvantages such as hypoxia and hypercapnia must be considered. Hypercapnia is one of the most frequent indications for converting to mOLV in cases of NITS, but the occurrence of permissive hypercapnia is a well-detailed and accepted side effect in NITS. It can be managed by re-expansion of the lung with intermittent non-invasive ventilation before the final indication for conversion occurs (18).

## Cardiac and Hemodynamic Effects of mOLV

As discussed above, the change in pressure in the thorax has a central role in the regulation of the lung and heart functions. During mechanical ventilation, the intrathoracic pressure and lung volume are increased, which has a negative effect on the atrial filling (preload) and cardiac output. This generally affects the right ventricle only; it does not concern the left ventricle if the patient has normal myocardial function (13). With the use of the NITS method, the preload can be increased compared to relaxed-surgery cases.

The difference between sOLV and mOLV can typically be observed when the thorax is just opened and the negative intrapleural pressure is lost. The development of positive intrapleural pressure during lung collapse causes hypoxic pulmonary vasoconstriction with increased pulmonary vascular resistance and diminished venous return. These changes should strain the right ventricle and cause a transient decrease in the ejection fraction of the right ventricle. In mOLV, if the patient is ventilated with positive pressure and PEEP, the hypoxic pulmonary vasoconstriction and pulmonary vascular resistance can be reduced, but in NITS, the opportunity to apply PEEP is very limited. If the surgical procedure can be interrupted for a short period to apply PEEP, it can reduce the hypoxic pulmonary vasoconstriction and pulmonary vascular resistance, but the administration of vasoconstrictive drugs is often required to stabilize cardiac output/function. For this reason, there are more frequent but transient cardiac instabilities only in this part of the NITS procedure, when the chest has just been opened. After this 5–8 min period of time, when the elevated hypoxic pulmonary vasoconstriction and pulmonary vascular resistance caused by the pressure change in the thorax cavity diminished, no difference in the cardiac and hemodynamic function had been observed between the mOLV and sOLV method, based on our experience. However, this kind of temporary instability can occur differently between epidural and paravertebral/intercostal + vagus nerve block methods used in NITS procedures (19–23). The positive hemodynamic effect of the epidural NITS method has previously been emphasized (20).

## Pathophysiological Effects of Locoregional Anesthesia in Nits

Thoracic epidural anesthesia (TEA) and paravertebral/intercostal anesthesia combined with vagus nerve block are the most frequently used locoregional anesthesia techniques in NITS. Some of the pathophysiological advantages of TEA are improved left ventricular function in coronary artery disease, decreased cardiac morbidity and mortality, fewer postoperative pulmonary complications, and adequate pain management (20, 22). However, there are limitations to the application of TEA, from spinal cord injury to epidural bleeding/hematoma and infection (24). Studies have explored the feasibility and advantages of paravertebral/intercostal + vagus nerve block over TEA, such as lower incidences of hypotension, pulmonary and urinary complications, and vomiting and nausea, but these studies have not mentioned any effect on cardiac function and pulmonary circulation (23, 25).

Another positive pathophysiological effect of TEA is its sympatholytic effect, which can also reduce surgical stress (20) and reduce troponin T and C-reactive protein (CRP) levels (26, 27). Moreover, in cases of mOLV during esophagectomy, TEA significantly reduced the levels of pro-inflammatory cytokines (interleukin [IL]-6 and IL-8) (28). Theoretically, this effect could be more effective in sOLV. Intercostal nerve block can also significantly reduce the stress response, e.g., reducing the levels of IL-6 and tumor necrosis factor α (29). In contrast, a meta-analysis showed no significant difference in postoperative inflammatory response (IL-6 and CRP levels) between the different types of anesthesia (30).

## Effects of Nits on Cancer

Only few studies have mentioned the short- and long-term effects of NITS on cancer behavior. One of the first findings on this topic was better survival after NITS compared to relaxed-surgery in cases of malignant pleural effusion (31). In a clinical study, better compliance with adjuvant chemotherapy with less toxicity after NITS lung lobectomy was verified, and 92% of patients who underwent NITS were able to receive the planned chemotherapy protocol, compared to 72% of relaxed-surgery cases (32). In another study, the overall survival and disease-free survival after lung cancer surgery were significantly better after sOLV than after mOLV, with type of anesthesia being an independent factor for both overall and disease-free survival in patients who had spontaneous ventilation (33). However, another study demonstrated no significant difference in disease recurrence and survival between the sOLV and mOLV methods (34). Meanwhile, a study on awake breast cancer surgery suggested that both extrathoracic cancer cases and lung cancer sOLV cases had shorter operative time and length of hospital stay (35, 36). Additionally, a case series presented about successful esophagectomy wherein the thoracic part was performed under spontaneous ventilation (37).

The role of cytokines in cancer regulation is well detailed in terms of molecular background (38, 39), especially IL-6, which has a central role and has been widely investigated in different clinical studies (5, 7, 40, 41). Cytokines can activate carcinogenesis and tumor growth and can protect cancer cells from therapy-induced gene damage and apoptosis (42). The levels of these released cytokines have been confirmed to be lower in sOLV cases than in mOLV cases, thus causing less damage on the anticancer activity of the body and promising better long-term results after NITS.

## Immune Effects of Nits mOLV and the Inflammatory Response

Surgical trauma and mOLV first induce a systemic inflammatory immune response; later, to compensate, a compensatory anti-inflammatory immune response is activated. Generally, cytokines are the key factors in the communication between cells taking part in the immune response and regulation of immune activity. The normal levels of cytokines have a positive effect on the defense mechanism; however, if their release exceeds the normal level, they cause negative side effects on immune regulation, inflammation, and the spread of cancer.

After surgery, an increased number of leukocytes and a reduced number of lymphocytes indicate deep immunosuppression; the lower number of lymphocytes is caused by postsurgical apoptosis (43). These changes in the levels of leukocytes, natural killer cells (44), lymphocytes, and cytokines have been investigated by several studies (40, 45) that showed that these changes were lower after NITS than in relaxed-surgery cases (31). The reduced inflammatory and immune changes after NITS suggest that immunosuppression is reduced after sOLV compared to relaxed-surgery cases (41, 45).

## Effects of Relaxant Drugs on Immune Function

The indirect effect of relaxation on the immune response in mOLV is detailed above, but the relaxant agents also affect the direct release of cytokines from macrophages. One experimental study implicated the presence of acetylcholine (ACh) and α7ACh receptors on blood mononuclear cells and the cholinergic anti-inflammatory pathway (46), showing that ACh significantly reduced the release of pro-inflammatory cytokines from human macrophages in culture. The drugs used to induce relaxation in mOLV block the neuromuscular junction by binding to the ACh receptors in combination with ACh. In theory, relaxants could block the ACh activity on macrophages; however, to our knowledge, whether relaxant drugs bind to ACh receptors on macrophages has not been demonstrated. It is likely that α7ACh receptors can be found on both the postsynaptic muscle membranes and the surfaces of macrophages. Therefore, relaxation has a double effect on the immune system: through mOLV-induced cytokine release and through the release of cytokines from macrophages. Both of these mechanisms can be avoided by performing NITS.

## Discussion

Performing NITS with spontaneous ventilation can prevent or reduce volutrauma in the alveoli that is caused by mOLV in relaxed-surgery cases. Due to the reduced pro-inflammatory response and release of fewer cytokines, NITS can moderate the immunosuppression caused by mechanical ventilation. During surgery with spontaneous ventilation, the V/Q match is better, which results in better oxygenation and cardiac output, as compared to relaxed-surgery cases. The reduced pro-inflammatory response and cytokine release can affect the central nervous system and the spread of cancer. The abovementioned pathophysiological advantages are the basis of the clinical observation that there are fewer complications after NITS lung surgery than in relaxant-surgery cases.

The limitation of this mini-review is that the advantages of NITS are discussed in the context of the negative effects of mOLV, which can be potentially reduced by performing NITS. However, some direct clinical and pathophysiological arguments (reduced inflammatory response, limited change in the number of leucocytes, and fewer postoperative morbidities) can support our theory that the NITS procedure is more physiological than mOLV. Survival in malignant pleural effusion (31) and lung cancer resection (33) improved more with NITS than with relaxed surgery, whereas compliance with adjuvant chemotherapy was better in patients who underwent NITS than in mOLV cases (32). It must be noted that the locoregional anesthesia in NITS is associated with a reduced cytokine release, contributing to a more physiological postoperative immune function.

However, there are only a few centers where the NITS procedure is routinely performed, and we could not find many prospective randomized studies about the direct advantages of sOLV. Furthermore, despite the effects of reduced surgical stress, the potential advantages of NITS are still not widely applied in daily clinical practice in Europe (47). Such hesitation is most likely due to the notion of “unsafe airway” being associated with the procedure by not only anesthesiologists but many thoracic surgeons. To minimize or prevent this airway problem, a new concept of the conversion method in NITS (48) and a new spontaneous ventilation procedure were introduced. In this new procedure, a short relaxation technique is applied for intubation with a double-lumen tube, and after that, the patient can breathe spontaneously. Here, the cough reflex is excluded by the vagus nerve block. In some cases, PEEP and pressure support ventilation as a means of breathing support are applied, but the ventilation periods are controlled by the patient (49, 50).

The problem remains the same with experimental studies on this topic. Therefore, the “NITS concept” needs improvement in at least three areas. First is adequate dissemination of information regarding this concept, such as provided by this special issue of the Journal. The discussion and interpretation of the current clinical experiences about sOLV and NITS could motivate non-NITS thoracic surgeons to apply this method. Second is the experimental background of the advantages of the spontaneous ventilation, specially sOLV. Both immunological and physiological aspects of the topic could be examined by animal and laboratory studies. Third is the possible improvement in the “NITS theory” by demonstration of its direct clinical impact on cancer. Currently, the most promising treatment for cancer is immunotherapy. According to the concept of immunotherapy, the more physiological the postoperative immune system is, the more effective is the anticancer function. However, according to the NITS theory, the immunosuppression is less after sOLV than after mOLV, suggesting that after NITS the postoperative immunotherapy should be more effective. The exploration of this question could be a topic of many further studies.

## Author Contributions

JL: writing the paper, collecting data, and conception of the manuscript. TN: collecting data. ZB: writing the paper and collecting data. ZS: conception of the anesthesiologic aspect of the topic. DP: conception of the immunological aspect of the topic. EV: conception of the manuscript. PH: conception and finalizing of the manuscript. All authors contributed to manuscript revision and approved the submitted version.

## Conflict of Interest

The authors declare that the research was conducted in the absence of any commercial or financial relationships that could be construed as a potential conflict of interest.

## Publisher's Note

All claims expressed in this article are solely those of the authors and do not necessarily represent those of their affiliated organizations, or those of the publisher, the editors and the reviewers. Any product that may be evaluated in this article, or claim that may be made by its manufacturer, is not guaranteed or endorsed by the publisher.

## References

[1] Finnerty CC Mabvuure NT Ali A Kozar RA Herndon DN. The surgically induced stress response. JPEN J Parenter Enter Nutr. (2013) 37(Supplement):21S−9. 10.1177/0148607113496117 24009246 PMC3920901

[2] Marik PE Flemmer M. The immune response to surgery and trauma: implications for treatment. J Trauma Acute Care Surg. (2012) 73:801–8. 10.1097/TA.0b013e318265cf87 22976420

[3] Misthos P Katsaragakis S Theodorou D Milingos N Skottis I. The degree of oxidative stress is associated with major adverse effects after lung resection: a prospective study. Eur J Cardiothorac Surg. (2006) 29:591–5. 10.1016/j.ejcts.2005.12.027 16476542

[4] Villamizar NR Darrabie MD Burfeind WR Petersen RP Onaitis MW Toloza E. Thoracoscopic lobectomy is associated with lower morbidity compared with thoracotomy. J Thorac Cardiovasc Surg. (2009) 138:419–25. 10.1016/j.jtcvs.2009.04.026 19619789

[5] Jones RO Anderson NH Murchison JT Brittan M Simon EJ Casali G. Innate immune responses after resection for lung cancer via video-assisted thoracoscopic surgery and thoracotomy. Innovations. (2014) 9:93–103; discussion 103. 10.1097/imi.0000000000000061 24755536

[6] Gonzalez-Rivas D Bonome C Fieira E Aymerich H Fernandez R Delgado M. Non-intubated video-assisted thoracoscopic lung resections: the future of thoracic surgery? Eur J Cardiothorac Surg. (2016) 49:721–31. 10.1093/ejcts/ezv136 25896196

[7] Jeon J Sung S Moon Y Koo J Hyun K Han K. Comparison of early postoperative cytokine changes in patients undergoing intubated and non-intubated thoracic surgery: a randomized controlled trial. Interact Cardiovasc Thorac Surg. (2021) 32:343–50. 10.1093/icvts/ivaa265 33831216 PMC8906678

[8] Gonzalez-Rivas D Fernandez R de la Torre M Rodriguez JL Fontan L Molina F. Single-port thoracoscopic lobectomy in a nonintubated patient: the least invasive procedure for major lung resection? Interact Cardiovasc Thorac Surg. (2014) 19:552–5. 10.1093/icvts/ivu209 25006214

[9] Kiss G Castillo M. Non-intubated anesthesia in thoracic surgery–technical issues. Ann Transl Med. (2015) 3:109. 10.3978/j.issn.2305-5839.2015.05.01 26046050 PMC4436424

[10] Lohser J Slinger P. Lung injury after one-lung ventilation: a review of the pathophysiologic mechanisms affecting the ventilated and the collapsed lung. Anesth Analg. (2015) 121:302–18. 10.1213/ANE.0000000000000808 26197368

[11] Lohser J. Chapter IS 5. Physiology of the lateral decubitus position, open chest and one-lung ventilation. In: Slinger P editor. Principles and Practice of Anesthesia for Thoracic Surgery. Vol. 71 (2011). p. 71–80. 10.1007/978-1-4419-0184-2_5

[12] Kozian A Schilling T Schütze H Senturk M Hachenberg T Hedenstierna G. Ventilatory protective strategies during thoracic surgery: effects of alveolar recruitment maneuver and low-tidal volume ventilation on lung density distribution. Anesthesiology. (2011) 114:1025–35. 10.1097/ALN.0b013e3182164356 21436678

[13] Shekerdemian L Bohn D. Cardiovascular effects of mechanical ventilation. Arch Dis Child. (1999) 80:475–80. 10.1136/adc.80.5.475 10208959 PMC1717913

[14] Jeon K Yoon JW Suh GY Kim J Kim K Yang M. Risk factors for post-pneumonectomy acute lung injury/acute respiratory distress syndrome in primary lung cancer patients. Anaesth Intensive Care. (2009) 37:14–9. 10.1177/0310057X0903700110 19157340

[15] Kozian A Schilling T Röcken C Breitling C Hachenberg T Hedenstierna G. Increased alveolar damage after mechanical ventilation in a porcine model of thoracic surgery. J Cardiothorac Vasc Anesth. (2010) 24:617–23. 10.1053/j.jvca.2009.09.016 20005130

[16] Kozian A Schilling T Fredén F Maripuu E Röcken C Strang C. One-lung ventilation induces hyperperfusion and alveolar damage in the ventilated lung: an experimental study. Br J Anaesth. (2008) 100:549–59. 10.1093/bja/aen021 18308740

[17] Putensen C Mutz NJ Putensen-Himmer G Zinserling J. Spontaneous breathing during ventilatory support improves ventilation–perfusion distributions in patients with acute respiratory distress Syndrome. Am J Respir Crit Care Med. (1999) 159:1241–8. 10.1164/ajrccm.159.4.9806077 10194172

[18] Kiss G Castillo M. Nonintubated anesthesia in thoracic surgery: general issues. Ann Transl Med. (2015) 3:110. 10.3978/j.issn.2305-5839.2015.04.21 26046051 PMC4436416

[19] Tacconi F Pompeo E Fabbi E Mineo TC. Awake video-assisted pleural decortication for empyema thoracis. Eur J Cardiothorac Surg. (2010) 37:594–601. 10.1016/j.ejcts.2009.08.003 19762250

[20] Mineo TC. Epidural anesthesia in awake thoracic surgery. Eur J Cardiothorac Surg. (2007) 32:13–9. 10.1016/j.ejcts.2007.04.004 17467287

[21] Hung MH Hsu HH Chan KC Chen KC Yie JC Cheng YJ. Non-intubated thoracoscopic surgery using internal intercostal nerve block, vagal block and targeted sedation. Eur J Cardiothorac Surg. (2014) 46:620–5. 10.1093/ejcts/ezu054 24585550

[22] Kao MC Lan CH Huang CJ. Anesthesia for awake video-assisted thoracic surgery. Acta Anaesthesiol Taiwan. (2012) 50:126–30. 10.1016/j.aat.2012.08.007 23026172

[23] Piccioni F Langer M Fumagalli L Haeusler E Conti B Previtali P. Thoracic paravertebral anaesthesia for awake video-assisted thoracoscopic surgery daily. Anaesthesia. (2010) 65:1221–4. 10.1111/j.1365-2044.2010.06420.x 20569246

[24] Freise H Van Aken HK. Risks and benefits of thoracic epidural anaesthesia. Br J Anaesth. (2011) 107:859–68. 10.1093/bja/aer339 22058144

[25] Davies RG Myles PS Graham JM. A comparison of the analgesic efficacy and side-effects of paravertebral vs epidural blockade for thoracotomy – a systematic review and meta-analysis of randomized trials. Br J Anaesth. (2006) 96:418–26. 10.1093/bja/ael020 16476698

[26] Loick HM Schmidt C Van Aken H Junker R Erren M Berendes E. High thoracic epidural anesthesia, but not clonidine, attenuates the perioperative stress response *via* sympatholysis and reduces the release of troponin T in patients undergoing coronary artery bypass grafting. Anesth Analg. (1999) 88:701–9. 10.1213/00000539-199904000-00001 10195508

[27] Palomero Rodríguez MA Suarez Gonzalo L Villar Alvarez F Varela Crespo C Moreno Gomez Limon I Criado Jimenez A. Thoracic epidural anesthesia decreases C-reactive protein levels in patients undergoing elective coronary artery bypass graft surgery with cardiopulmonary bypass. Minerva Anestesiol. (2008) 74:619–26. 18971890

[28] Fares KM Mohamed SA Hamza HM Sayed DM Hetta DF. Effect of thoracic epidural analgesia on pro-inflammatory cytokines in patients subjected to protective lung ventilation during Ivor Lewis esophagectomy. Pain Phys. (2014) 17:305–15. Erratum in: *Pain Phys* (2014) 17:475. 10.36076/ppj.2014/17/305 25054390

[29] Zhan Y Chen G Huang J Hou B Liu W Chen S. Effect of intercostal nerve block combined with general anesthesia on the stress response in patients undergoing minimally invasive mitral valve surgery. Exp Ther Med. (2017) 14:3259–64. 10.3892/etm.2017.4868 28912876 PMC5585755

[30] Alhayyan A McSorley S Roxburgh C Kearns R Horgan P McMillan D. The effect of anesthesia on the postoperative systemic inflammatory response in patients undergoing surgery: a systematic review and meta-analysis. Surg Open Sci. (2020) 2:1–21. 10.1016/j.sopen.2019.06.001 32754703 PMC7391900

[31] Mineo TC Ambrogi V. Immune effects after uniportal nonintubated video-thoracoscopic operations. Video-assist. J Thorac Surg. (2018) 3:4–10. 10.21037/vats.2018.01.02

[32] Furák J Paróczai D Burián K Szabó Z Zombori T. Oncological advantage of nonintubated thoracic surgery: better compliance of adjuvant treatment after lung lobectomy. Thorac Cancer. (2020) 11:3309–16. 10.1111/1759-7714.13672 32985138 PMC7606006

[33] Zheng J Liang H Wang R Zhong R Jiang S Wang W. Perioperative and long-term outcomes of spontaneous ventilation video-assisted thoracoscopic surgery for non-small cell lung cancer. Transl Lung Cancer Res. (2021) 10:3875–87. 10.21037/tlcr-21-629 34858778 PMC8577985

[34] Wang ML How CH Hung MH Huang HH Hsu HH Cheng YJ Chen JS. Long-term outcomes after nonintubated versus intubated thoracoscopic lobectomy for clinical stage I non-small cell lung cancer: A propensity-matched analysis. J Formos Med Assoc. (2021) 120:1949–56. 10.1016/j.jfma.2021.04.018 33994233

[35] Vanni G Pellicciaro M Materazzo M Dauri M D'angelillo RM Buonomo C. Awake breast cancer surgery: strategy in the beginning of COVID-19 emergency. Breast Cancer. (2021) 28:137–44. 10.1007/s12282-020-01137-5 32734327 PMC7391474

[36] Santonastaso DP de Chiara A Russo E Gamberini E Lucchi L Sibilio A. A possible future for anaesthesia in breast surgery: thoracic paravertebral block and awake surgery. A prospective observational study. Tumori. (2021) 107:125–31. 10.1177/0300891620951626 32842912

[37] Xu Q Mo X Xiong J Zhang Y. Case report: discontinuous spontaneous ventilating anesthesia for McKeown esophagectomy by laryngeal mask: a case series-A novel approach of discontinuous spontaneous ventilating anesthesia for esophagectomy. Front Surg. (2021) 8:783859. 10.3389/fsurg.2021.783859 34957206 PMC8696255

[38] Dunlop RJ Campbell CW. Cytokines and advanced cancer. J Pain Symptom Manage. (2000) 20:214–32. 10.1016/S0885-3924(00)00199-8 11018340

[39] Negus RP Balkwill FR. Cytokines in tumour growth, migration and metastasis. World J Urol. (1996) 14:157–65. 10.1007/BF00186895 8806194

[40] Breunig A Gambazzi F Beck-Schimmer B Tamm M Lardinois D Oertli D. Cytokine and chemokine response in the lungs, pleural fluid and serum in thoracic surgery using one-lung ventilation. J Inflamm. (2011) 8:32. 10.1186/1476-9255-8-32 22078633 PMC3253056

[41] Mineo TC Sellitri F Vanni G Gallina FT Ambrogi V. Immunological and inflammatory impact of non-intubated lung metastasectomy. Int J Mol Sci. (2017) 18:1466. 10.3390/ijms18071466 28686211 PMC5535957

[42] Briukhovetska D Dörr J Endres S Libby P Dinarello CA Kobold S. Interleukins in cancer: from biology to therapy. Nat Rev Cancer. (2021) 21:481–99. 10.1038/s41568-021-00363-z 34083781 PMC8173513

[43] Dabrowska AM Słotwiński R. The immune response to surgery and infection. Cent Eur J Immunol. (2014) 39:532–7. 10.5114/ceji.2014.47741 26155175 PMC4439968

[44] Vanni G Tacconi F Sellitri F Ambrogi V Mineo TC Pompeo E. Impact of awake videothoracoscopic surgery on postoperative lymphocyte responses. Ann Thorac Surg. (2010) 90:973–8. 10.1016/j.athoracsur.2010.04.070 20732526

[45] Yu MG Jing R Mo YJ Lin F Du XK Ge WY. Non-intubated anesthesia in patients undergoing video-assisted thoracoscopic surgery: a systematic review and meta-analysis. PLoS ONE. (2019) 14:e0224737. 10.1371/journal.pone.0224737 31714904 PMC6850529

[46] Borovikova LV Ivanova S Zhang M Yang H Botchkina GI Watkins LR. Vagus nerve stimulation attenuates the systemic inflammatory response to endotoxin. Nature. (2000) 405:458–62. 10.1038/35013070 10839541

[47] Tosi D Nosotti M Bonitta G Mendogni P Bertolaccini L Spaggiari L. Anatomical segmentectomy versus pulmonary lobectomy for stage I non-small-cell lung cancer: patients selection and outcomes from the European Society of Thoracic Surgeons database analysis. Interact Cardiovasc Thorac Surg. (2021) 32:546–51. 10.1093/icvts/ivaa298 33313840 PMC8906695

[48] Furák J Szabó Z Tánczos T Paszt A Rieth A Németh T. Conversion method to manage surgical difficulties in non-intubated uniportal video-assisted thoracic surgery for major lung resection: simple thoracotomy without intubation. J Thorac Dis. (2020) 12:2061–9. 10.21037/jtd-19-3830 32642108 PMC7330381

[49] Furák J Szabó Z. Spontaneous ventilation combined with double-lumen tube intubation in thoracic surgery. Gen Thorac Cardiovasc Surg. (2021) 69:976–82. 10.1007/s11748-020-01572-3 33433769

[50] Furák J Barta Z Lantos J Ottlakán A Németh T Pécsy B. Better intraoperative cardiopulmonary stability and similar postoperative results of spontaneous ventilation combined with intubation than non-intubated thoracic surgery. Gen Thorac Cardiovasc Surg. (2022) 5. 10.1007/s11748-021-01768-1. [Epub ahead of print]. 34985733

